# The complete chloroplast genome sequence of *Anthurium andraeanum* Linden (Araceae; Pothoideae)

**DOI:** 10.1080/23802359.2023.2185081

**Published:** 2023-03-08

**Authors:** Xiao Wan, Yaying Ge, Gangmin Pan, Danqing Tian

**Affiliations:** Zhejiang Institute of Landscape Plants and Flowers, Zhejiang Academy of Agricultural Sciences, Hangzhou, China

**Keywords:** *Anthurium andraeanum*, chloroplast genome, phylogeny, pothoideae

## Abstract

The chloroplast genome of *Anthurium andraeanum* Linden 1877 was assembled and analyzed in this study. The genome size is 162,560 bp, of which contains a large single-copy (LSC) region with 88,814 bp, a small single-copy (SSC) region with 22,856 bp, and two inverted repeat regions (IRA and IRB) with 25,445 bp, respectively. The plastome contains 124 genes, including 80 protein-coding genes, 37 tRNAs, six rRNAs and one pseudogene. Phylogenetic analysis indicated that *A. andraeanum* is a member of Pothoideae and sister to *A. huixtlense*.

## Introduction

**Figure 1.**
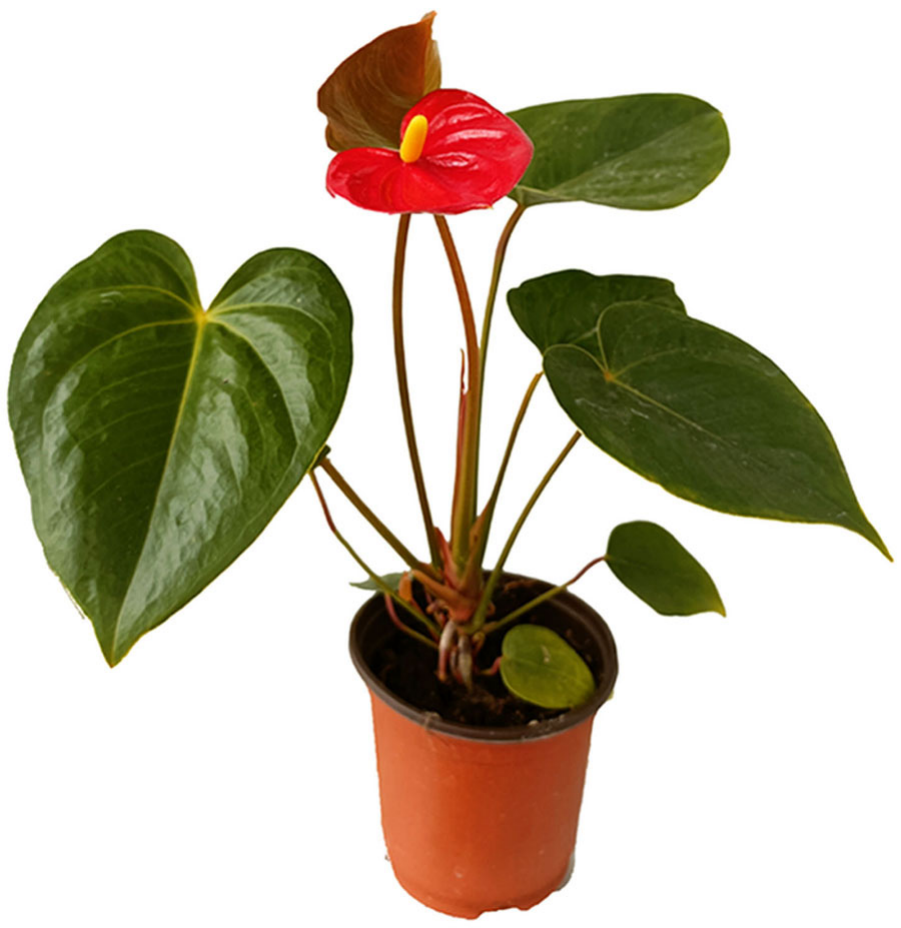
Potted *Anthurium andraeanum* ‘Alabama’. The species reference image was taken by Xiao Wan.

*Anthurium* Schott is the largest genus of Araceae, including approximately 950 species distributed in the neotropics (Boyce and Croat 2011, continuously updated; Carlsen and Croat 2013; Poli et al. 2017). Due to brilliant flower color and (or) fine reticulate venation in leaf, many species have been cultivated as ornamental houseplants and cut flowers since the nineteenth century (Brown 2000). *A. andraeanum*, occupying the second place in tropical flower trade in this world, is the most popular use in crossbreeding of the genus. However, the complete chloroplast genome of this species has not been reported as yet. Herein, we assembled and annotated a complete plastid genome of *A. andraeanum* (Figure 1).

## Materials and methods

Mature leaf samples were collected from an individual of *A. andraeanum* ‘Alabama’ (Figure 1) planted in the greenhouse of Zhejiang Institute of Landscape Plants and Flowers, Hangzhou, China (120°13′5″E, 30°3′41″N). A voucher specimen (HZ0711) of the sampling plant was deposited in the herbarium of Zhejiang Academy of Agricultural Sciences, Hangzhou, China (Xiao Wan, wanxiaoww@163.com). Chloroplasts were isolated from the leaf samples with the modified percoll density gradient centrifugation method (Chen and Chen 2014) then the plastid genomic DNA was extracted using Hlingene plant DNA extraction kit (production code: NG412S) following the manufacturer procedure. Qualified DNA was sequenced in an Illumina HiSeq 2000 platform (Illumina, CA, USA). The clean reads were used to assemble a completed chloroplast genome with software GetOrganelle (Jin et al. 2020) with ‘embplant_pt’ as reference. The assembled results were annotated using GeSeq (Tillich et al. 2017) combined with ‘BLAT search’, ‘Hmmer profile search’, and ‘tRNAscan-SE v2.0.7’. One of assemblies contained a SSC with correct orientation was picked as the authentic chloroplast genome. Structure of genes that are difficult to annotate was identified and illustrated using cpgview (http://www.1kmpg.cn/cpgview) (Liu et al. 2023). The correct assembled genome was further assessed with sequencing data using BAMstats (https://bamstats.sourceforge.net/) with default parameters and Integrative Genomics Viewer (Robinson et al. 2017) with a tdf-formatted file. The genome sequence can be assessed on figshare (https://doi.org/10.6084/m9.figshare.21687575.v1) and NCBI (https://www.ncbi.nlm.nih.gov/) with GenBank accession OP938256. Phylogenetic relationships among *A. andraeanum* and its relatives in Araceae family were reconstructed using IQ-TREE v1.6.12 (Nguyen et al. 2015) with 1000 bootstrap replicates.

## Results and discussion

**Figure 2.**
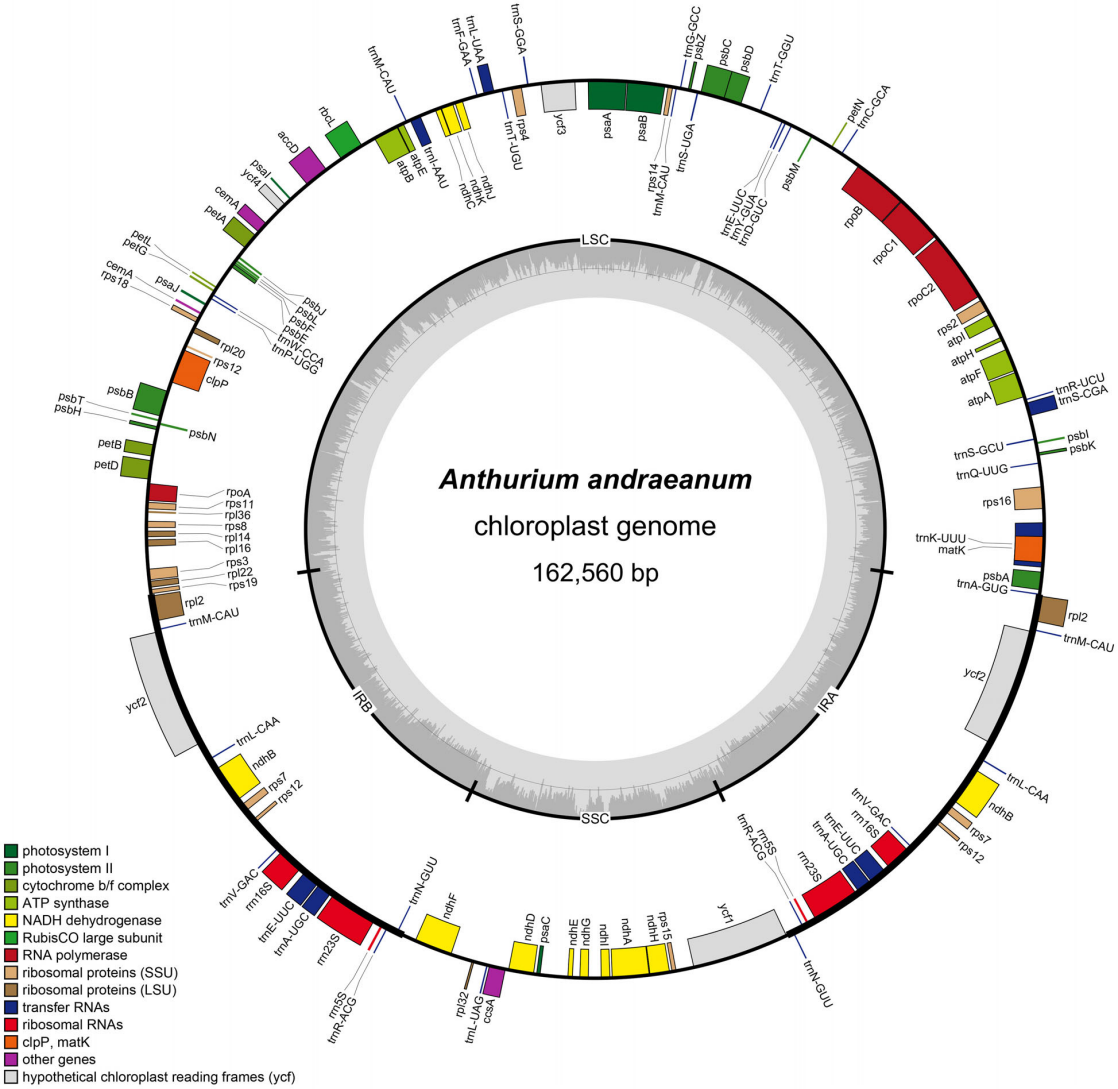
The circular map of the chloroplast genome of *Anthurium andraeanum.* LSC, SSC, and IR represent large single-copy, small single-copy and inverted repeat regions respectively. The gene in the loop represent the clockwise direction of transcription, and the genes outside the loop represent the anti-clockwise direction of transcription. The genes with various functions were labeled using different colors. Built-in gray histogram showed the GC content of the genome, and the gray line in middle represented the threshold of 50%.

The newly assembled plastid genome of *A. andraeanum* is 162,560 bp in length. The sequencing coverage depths of the genome ranged from 12× to 640× and the mean coverage depth is 79.6×, which indicated that the genome assembly is reliable (Supplementary Figure 1). The genome contains a large single-copy (LSC) region with 88,814 bp, a small single-copy (SSC) region with 22,856 bp, and two inverted repeat regions (IRA and IRB) with 25,445 bp (Figure 2). A total of 124 genes, including 80 protein-coding genes, 37 tRNAs, six rRNAs and one pseudogene, were annotated in this plastome (Table 1). There are 13 protein-coding genes containing intron(s) and one trans-splicing genes *rps12* (Supplementary Figure 2). In this study, we found the number of protein-coding genes and tRNA of *A. andraeanum* were different from those of a congener *A. huixtlense.* The plastid genome of *A. huixtlense* contains 85 protein-coding genes and eight tRNAs (Abdullah et al. 2020). The difference between the plastid genomes is listed in Table 2.

**Table 1.** Genes in *Anthurium andraeanum* chloroplast genome.

Function	Family name	List of genes
Photosynthesis	Subunits of ATP synthase	*atpA*, *atpB*, *atpE*, *atpF*, *atpH*, *atpI*
	ATP-dependent Clp protease proteolytic subunit	*clpP*
	Subunits of photosystem II	*psbA*, *psbB*, *psbC*, *psbD*, *psbE*, *psbF*, *psbH*, *psbI*, *psbJ*, *psbK*, *psbL*, *psbM*, *psbN*, *psbT*, *psbZ*
	Subunits of NADH-dehydrogenase	*ndhA*, *ndhB*, *ndhB*, *ndhC*, *ndhE*, *ndhF*, *ndhG*, *ndhH*, *ndhI*, *ndhJ*, *ndhK*
	Subunits of cytochrome b/f complex	*petA*, *petB*, *petD*, *petG*, *petL*, *petN*
	Subunits of photosystem I	*psaA*, *psaB*, *psaC*, *psaI*, *psaJ*
	Photosystem I assembly	*ycf3*, *ycf4*
	Subunit of rubisco	*rbcL*
Self-replication	Large subunit of ribosome	*rpl2*, *rpl2*, *rpl14*, *rpl16*, *rpl20*, *rpl22*, *rpl32*, *rpl36*
	DNA dependent RNA polymerase	*rpoA*, *rpoB*, *rpoC1*, *rpoC2*
	Small subunit of ribosome	*rps2*, *rps3*, *rps4*, *rps7*, *rps7*, *rps8*, *rps11*, *rps12*, *rps14*, *rps15*, *rps16*, *rps18*, *rps19*
	rRNA genes	*rrn5S*, *rrn5S*, *rrn16S*, *rrn16S*, *rrn23S*, *rrn23S*
	tRNA genes	*trnA-GUG*, *trnA-UGC*, *trnA-UGC*, *trnC-GCA*, *trnD-GUC*, *trnE-UUC*, *trnE-UUC*, *trnE-UUC*, *trnF-GAA*, *trnG-GCC*, *trnI-AUU*, *trnK-UUU*, *trnL-CAA*, *trnL-CAA*, *trnL-UAA*, *trnL-UAG*, *trnM-CAU*, *trnM-CAU*, *trnM-CAU*, *trnM-CAU*, *trnN-GUU*, *trnN-GUU*, *trnP-UGG*, *trnQ-UUG*, *trnR-ACG*, *trnR-ACG*, *trnR-UCU*, *trnS-CGA*, *trnS-GCU*, *trnS-GGA*, *trnS-UGA*, *trnT-GGU*, *trnT-UGU*, *trnV-GAC*, *trnV-GAC*, *trnW-CCA*, *trnY-GUA*
Other genes	Subunit of Acetyl-CoA-carboxylase	*accD*
	c-type cytochrom synthesis gene	*ccsA*
	Envelop membrane protein	*cemA*, *cemA*
	Maturase	*matK*
Pseudogene	Subunits of NADH-dehydrogenase	*ndhD*
Unkown	Conserved open reading frames	*ycf1*, *ycf2*, *ycf2*

**Table 2.** Difference of chloroplast genes between *Anthurium andraeanum* and *A. huixlense.*

	*A. andraeanum*	*A. huixlense*
Protein-coding gene	cemA (2)*, rpl33	cemA (1), InfA, ndhD rpl23 (2)
Pseudogenes	ndhD	—*
tRNA	trnA (3), trnE (3), trnI (1), trnV (1)	trnA(2), trnE (1), trnH, trnI (2), trnV (3)
rRNA	—	rrn4.5S (2)

*Brackets indicate the gene numbers and—indicate no comparative item in chloroplast genomes.

**Figure 3.**
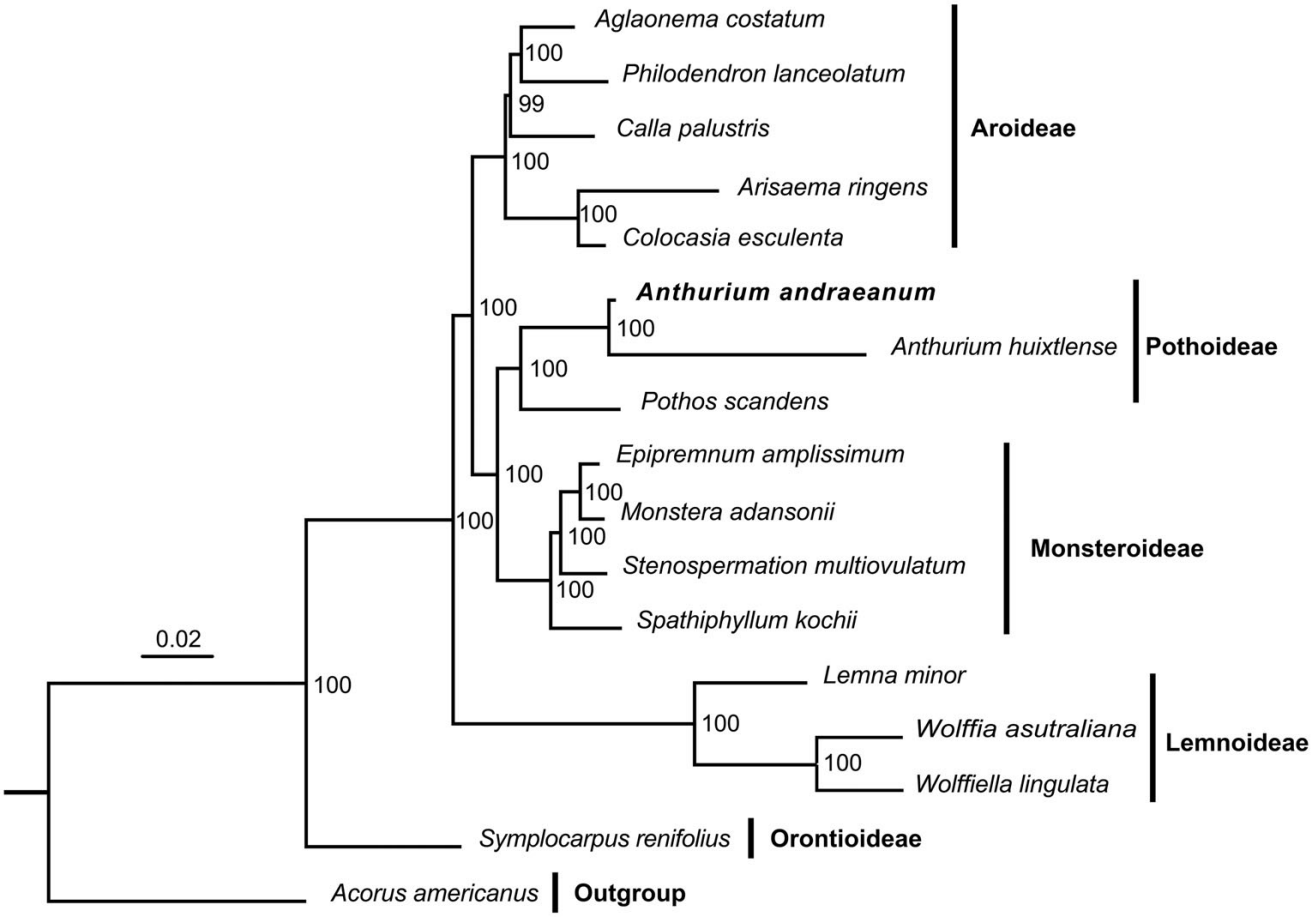
Maximum likelihood phylogenetic tree reconstructed with IQ-TREE based on complete chloroplast genome sequences from *A. andraeanum* and 17 other species of Araceae. *Acorus americanus* (EU273602.1), *Aglaonema costatum* (MN046881.1), *Anthurium huixtlense* (NC_051870.1), *Arisaema ringens* (MK111107.1), *Calla palustris* (MN046887.1), *Colocasia esculenta* (JN105690.1), *Epipremnum amplissimum* (MN477424.1), *Lemna minor* (DQ400350.1), *Monstera adansonii* (MN046888.1), *Philodendron lanceolatum* (MN551187.1), *Pothos scandens* (MN046891.1), *Spathiphyllum kochii* (NC_030371.1), *Stenospermation multiovulatum* (MN046893.1), *Symplocarpus renifolius* (KY039276.1), *Wolffia australiana* (JN160605.1), and *Wolffiella lingulata* (NC_015894.1).

Interestingly, we found that two *rpl2* genes in the inverted repeat regions might use an ‘AUA’ initiation codon instead of traditional one ‘AUG’ in *A. andraeanum* plastid genome. To date, ‘AUA’ was only accepted as an alternative start codon in gene *rpoA* from single-celled plant *Eutreptiella pomquetensis* (Dabbagh et al. 2017). Hence, ‘AUA’ initiation codon in the higher plants still needs to be verified. In addition, to confirm the phylogenetics of *Anthurium andraeanum*, a phylogenetic tree based on the principle of maximum likelihood estimate was reconstructed with other 16 representative species of Araceae (Figure 3). The best model GTR + F+R4 was selected for the phylogenetic reconstruction with IQTREE. The ML phylogenetic tree indicated that *A. andraeanum* is a member of Pothoideae and sister to *A. huixtlense*. In Pothoideae cluster, *Anthurium* species are sister to genus *Pothos*, which is consistent with a previous study (Abdullah et al. 2020).

In conclusion, our study presents a complete chloroplast genome sequence of *Anthurium andraeanum*, an important and popular horticultural plant. We described the sequence structures and annotated genes in the genome. Our work provides important data for understanding phylogenetics of *A. andraeanum* and development of molecular markers for the species.
